# Correction: SKLB023 as an iNOS inhibitor alleviated liver fibrosis by inhibiting the TGF-beta/Smad signaling pathway

**DOI:** 10.1039/d6ra90084d

**Published:** 2026-08-04

**Authors:** Jinhang Zhang, Yanping Li, Qinhui Liu, Rui Li, Shiyun Pu, Lina Yang, Yanhuan Feng, Liang Ma

**Affiliations:** a Laboratory of Clinical Pharmacy and Adverse Drug Reaction, Department of Pharmacy, Collaborative Innovation Centre of Biotherapy, West China Hospital of Sichuan University Chengdu 610041 China; b Kidney Research Lab, Division of Nephrology, West China Hospital of Sichuan University Chengdu 610041 China Liang_m@scu.edu.cn +86-28-85423341 +86-28-85164167

## Abstract

Correction for ‘SKLB023 as an iNOS inhibitor alleviated liver fibrosis by inhibiting the TGF-beta/Smad signaling pathway’ by Jinhang Zhang *et al.*, *RSC Adv.*, 2018, **8**, 30919–30924, https://doi.org/10.1039/c8ra04955f.

The authors regret that in the originally published manuscript, the H&E and Oil Red O staining images for the control group in Fig. 2E were inadvertently misapplied. The corrected Fig. 2E is shown here. This correction does not affect the results and conclusions of the study. The authors sincerely apologize for any inconvenience this may have caused.

**Fig. 1:**
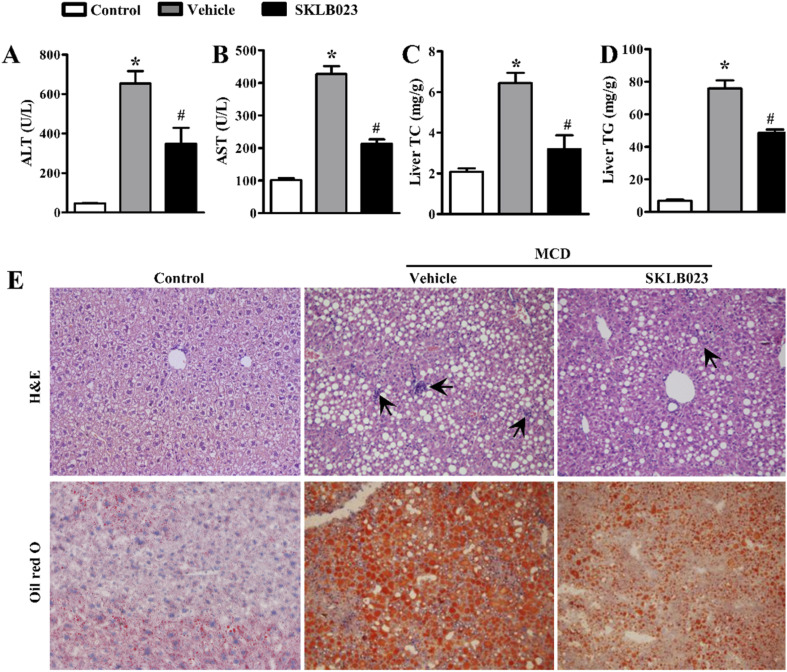
SKLB023 alleviates steatosis in MCD diet-induced mice. Serum levels of (A) ALT and (B) AST; hepatic levels of (C) total cholesterol (TC) and (D) triglyceride (TG); and (E) representative H&E staining and Oil Red O staining of liver sections of mice with indicated treatments. *P < 0.05 *versus* the control group, ^#^*P* < 0.05 *versus* the vehicle, *n* = 6 for each group.

The Royal Society of Chemistry apologises for these errors and any consequent inconvenience to authors and readers.

